# Influence of Hyperglycemia and Diabetes on Cardioprotection by Humoral Factors Released after Remote Ischemic Preconditioning (RIPC)

**DOI:** 10.3390/ijms22168880

**Published:** 2021-08-18

**Authors:** Carolin Torregroza, Lara Gnaegy, Annika Raupach, Martin Stroethoff, Katharina Feige, André Heinen, Markus W. Hollmann, Ragnar Huhn

**Affiliations:** 1 Department of Anesthesiology, Medical Faculty and University Hospital Duesseldorf, Heinrich-Heine-University Duesseldorf, Moorenstr. 5, 40225 Duesseldorf, Germany; Carolin.Torregroza@med.uni-duesseldorf.de (C.T.); l.gnaegy@gmx.net (L.G.); Martin.Stroethoff@med.uni-duesseldorf.de (M.S.); KatharinaKristina.Feige@med.uni-duesseldorf.de (K.F.); Ragnar.Huhn@med.uni-duesseldorf.de (R.H.); 2 Institute of Cardiovascular Physiology, Medical Faculty and University Hospital Duesseldorf, Heinrich-Heine-University Duesseldorf, Universitaetsstr. 1, 40225 Duesseldorf, Germany; Andre.Heinen@uni-duesseldorf.de; 3 Department of Anesthesiology, Amsterdam University Medical Center (AUMC), Location AMC, Meiberdreef 9, 1105 AZ Amsterdam, The Netherlands; M.W.Hollmann@amsterdamumc.nl

**Keywords:** diabetes mellitus, humoral factor, hyperglycemia, myocardial infarction, remote ischemic preconditioning

## Abstract

Remote ischemic preconditioning (RIPC) protects hearts from ischemia–reperfusion (I/R) injury in experimental studies; however, clinical RIPC trials were unsatisfactory. This discrepancy could be caused by a loss of cardioprotection due to comorbidities in patients, including diabetes mellitus (DM) and hyperglycemia (HG). RIPC is discussed to confer protective properties by release of different humoral factors activating cardioprotective signaling cascades. Therefore, we investigated whether DM type 1 and/or HG (1) inhibit the release of humoral factors after RIPC and/or (2) block the cardioprotective effect directly at the myocardium. Experiments were performed on male Wistar rats. Animals in part 1 of the study were either healthy normoglycemic (NG), type 1 diabetic (DM1), or hyperglycemic (HG). RIPC was implemented by four cycles of 5 min bilateral hind-limb ischemia/reperfusion. Control (Con) animals were not treated. Blood plasma taken in vivo was further investigated in isolated rat hearts in vitro. Plasma from diseased animals (DM1 or HG) was administered onto healthy (NG) hearts for 10 min before 33 min of global ischemia and 60 min of reperfusion. Part 2 of the study was performed vice versa—plasma taken in vivo, with or without RIPC, from healthy rats was transferred to DM1 and HG hearts in vitro. Infarct size was determined by TTC staining. Part 1: RIPC plasma from NG (NG Con: 49 ± 8% vs. NG RIPC 29 ± 6%; *p* < 0.05) and DM1 animals (DM1 Con: 47 ± 7% vs. DM1 RIPC: 38 ± 7%; *p* < 0.05) reduced infarct size. Interestingly, transfer of HG plasma showed comparable infarct sizes independent of prior treatment (HG Con: 34 ± 9% vs. HG RIPC 35 ± 9%; ns). Part 2: No infarct size reduction was detectable when transferring RIPC plasma from healthy rats to DM1 (DM1 Con: 54 ± 13% vs. DM1 RIPC 53 ± 10%; ns) or HG hearts (HG Con: 60 ± 16% vs. HG RIPC 53 ± 14%; ns). These results suggest that: (1) RIPC under NG and DM1 induces the release of humoral factors with cardioprotective impact, (2) HG plasma might own cardioprotective properties, and (3) RIPC does not confer cardioprotection in DM1 and HG myocardium.

## 1. Introduction

Diabetes mellitus (DM) is one of the main comorbidities associated with cardiovascular diseases and occurrence of myocardial infarction [1]. In 2019, up to 463 million people worldwide were suffering from a diabetic condition and its deleterious consequences [2]. Besides a higher overall mortality in these patients [3], diabetes mellitus, along with acute hyperglycemia, is considered an independent risk factor for the development of myocardial infarction and ischemic heart disease. Further, patients with diabetes mellitus are also more likely to suffer from major perioperative adverse cardiac events compared to the nondiabetic population [4]. Interestingly, hyperglycemia seems to be more than just an accompanying bystander of diabetic conditions [5]. Elevated blood glucose levels are not only present in patients with diagnosed diabetes or chronic metabolic syndrome [6]; they also frequently appear in nondiabetic individuals as a metabolic response to stress conditions, such as the perioperative setting [5]. Furthermore, hyperglycemia has been shown to directly correlate with increased morbidity and mortality in patients, independent of a pre-existing diabetic disease [7]. Due to a rising incidence of patients suffering from diabetes mellitus and hyperglycemia, an increase in the occurrence of myocardial infarction and ischemic heart disease is to be expected.

Coronary revascularization after myocardial infarction, along with resupply of oxygen to the myocardium, is crucial for patient survival. Paradoxically, during reperfusion, a cascade of complex cellular processes is triggered by restored blood supply, including electrolyte shift, as well as release of intracellular enzymes and proapoptotic factors, resulting in cardiomyocyte damage and death [8]. This phenomenon of myocardial damage due to restored coronary perfusion is called ischemia/reperfusion (I/R) injury and accounts for up to 50% of the final infarct size [9]. Hence, I/R injury is one major aspect for poor outcome in patients suffering from myocardial infarction. Unfortunately, the challenge in protecting the heart against I/R injury in the clinical setting has, as yet, failed to be resolved.

Given the poor prognosis of these patients and the overall burden to the global health system, developing new treatment strategies to improve survival after myocardial infarction is of paramount importance. A noninvasive and clinically practical technique is the concept of remote ischemic preconditioning (RIPC) [10]. Transient ischemia of remote organs or tissues—such as hind-limb ischemia via blood pressure cuffs—confers cardioprotective properties against I/R injury. Release of humoral factors into the bloodstream targeting known myocardial signaling cascades, such as reperfusion injury salvage kinase (RISK) pathway, is believed to induce cardioprotection by RIPC [11]. While the infarct size reducing effect of RIPC has been demonstrated in numerous experimental studies, translation into the clinical setting remains unsatisfactory [12]. Few studies have shown potential benefits of RIPC [13]; however, recent large multicenter trials were unable to detect any improvement in patient outcome after RIPC maneuver [14,15]. A clear rationale for this discrepancy has not been determined conclusively, but confounding factors, such as comedication, anesthetic regimen, age, or comorbidities of respective patients, are discussed [12]. As the patient population in clinical trials usually presents a vast heterogeneity, protective effects of RIPC for certain patient groups could be masked by loss of cardioprotection from another cohort. Hence, identifying those patients possibly benefitting from RIPC maneuver should be of particular concern. Referring to diabetes mellitus and hyperglycemia, both experimental and clinical studies have indicated a loss of cardioprotection by ischemic [16] and pharmacological [17] stimuli under these conditions.

A possible loss of protection by RIPC under confounding factors, such as diabetes mellitus or hyperglycemia, could be caused by impairment—release or transfer—of humoral factors. Another alternative is structural changes of the diseased myocardium itself under diabetic or hyperglycemic conditions, resulting in blocked effectiveness of RIPC.

Therefore, we returned from bed to benchside to determine whether diabetic or hyperglycemic conditions negatively influence cardioprotection by RIPC. Our primary aim was to investigate whether the loss of protective properties results from the diseased myocardium itself or due to an impaired release of humoral factors. In order to differentiate between the influence on humoral factors and the myocardium, we employed a translational approach, transferring plasma taken in vivo onto isolated hearts in vitro.

## 2. Results

### 2.1. Animal Characteristics and Glucose Values

For each part of the study, no differences in characteristics were shown between control (Con) and RIPC animals within each group (Table 1, Tables S1 and S2, see Supplementary Materials). Blood glucose values were measured immediately before plasma sampling in vivo after RIPC or Con treatment. Healthy, normoglycemic (NG) animals included in this study had glucose values of 124 ± 14 mg/dL for Con and 123 ± 31 mg/dL for RIPC, respectively. Diabetes mellitus type 1 (DM1) animals in part 1 and 2 of the study had hyperglycemic blood glucose levels (part 1: DM1 Con 540 ± 58 mg/dL vs. DM1 RIPC 447 ± 66 mg/dL, ns) and (part 2: DM1 Con 495 ± 87 mg/dL vs. DM1 RIPC 481 ± 82 mg/dL, ns), respectively. For all DM1 animals, hyperglycemia was achieved one week after streptozotocin application and remained stable throughout the following 3 weeks. Referring to hyperglycemia (HG) groups, glucose levels showed no difference between Con and RIPC groups before plasma sampling in vivo (HG Con 543 ± 69 mg/dL vs. HG RIPC 531 ± 63 mg/dL, ns), or between Con and RIPC groups during in vitro experiments taken from coronary effluent (HG Con 433 ± 13 mg/dL vs. HG RIPC 442 ± 35 mg/dL, ns). Con HG animals had significantly higher glucose values than DM1 RIPC animals (HG Con 543 ± 69 mg/dL vs. DM1 RIPC 447 mg/dL, *p* = 0.0371). However, comparing RIPC and Con animals between HG and DM1 groups, no statistical differences were detected (HG RIPC 531 ± 63 vs. DM1 RIPC 447 ± 66, and HG Con 543 ± 69 vs. DM1 Con 540 ± 58, both ns.).

For both part 1 and 2, a total of 100 animals were included in in vitro experiments. Hearts of 21 animals were excluded from statistical analysis due to not meeting required hemodynamic baseline values. As plasma from one in vivo animal was only used for one in vitro experiment, 100 animals were included in the in vivo protocol and plasma sampling. Mortality rate for these experiments was 7%, which was caused by difficulties in intubation and cannulation of the jugular vein and/or carotid artery.

### 2.2. Infarct Size Measurements

**Figure 1:**
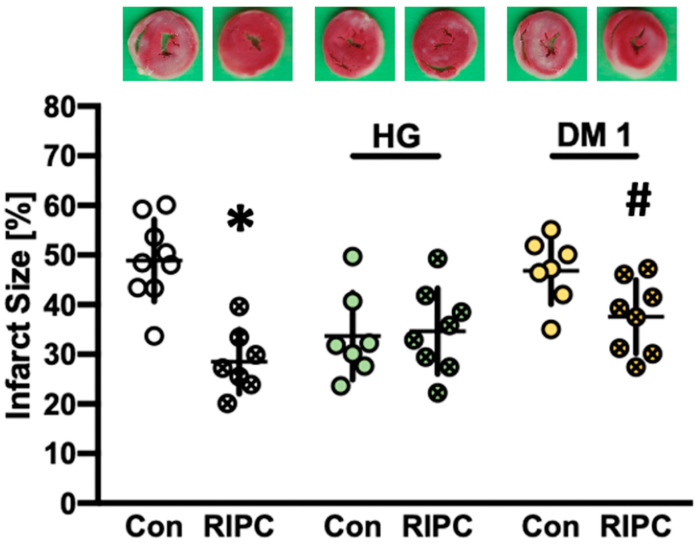
Infarct size measurement of part 1, including representative photos of each group. Con = control; RIPC = remote ischemic preconditioning; HG = hyperglycemia; DM1 = diabetes mellitus type 1. Data are presented as mean ± SD. * *p* < 0.0001 vs. Con, ^#^ *p* = 0.025 vs. DM1 Con.

The infarct sizes from part 1 are shown in Figure 1. Preconditioning with plasma from normoglycemic RIPC animals significantly reduced infarct size in healthy hearts compared to plasma from animals without RIPC treatment (NG RIPC: 29 ± 6% vs. NG Con: 49 ± 8%, *p* < 0.0001). Furthermore, transfer of RIPC plasma from diabetic hearts (DM1) onto naïve hearts also induced a significant infarct size reduction (DM1 RIPC: 38 ± 7% vs. DM1 Con: 47 ± 7%, *p* = 0.025). Interestingly, hearts treated with plasma from hyperglycemic (HG) animals showed infarct sizes similar to known protective stimuli. However, this effect could not be enhanced further by additional RIPC treatment (HG Con: 34 ± 9% vs. HG RIPC: 35 ± 9%, ns.).

**Figure 2:**
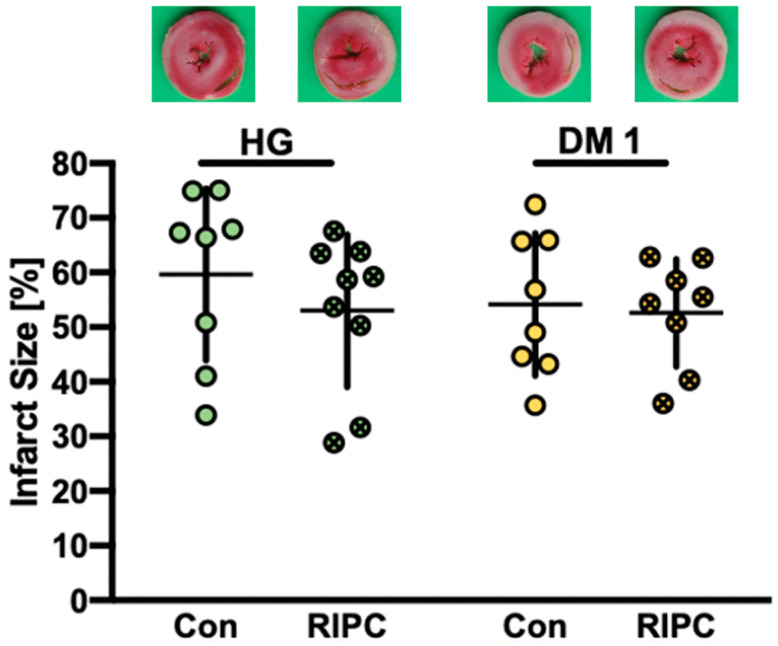
Infarct size measurement of part 2, including representative photos of each group. Con = control; RIPC = remote ischemic preconditioning; HG = hyperglycemia; DM1 = diabetes mellitus type 1. Data are presented as mean ± SD.

Results from part 2 infarct size measurements are displayed in Figure 2. Plasma taken from normoglycemic animals with (NG RIPC) or without RIPC (NG Con) treatment had no impact on infarct size in HG hearts (HG Con: 60 ± 16% vs. HG RIPC: 53 ± 14%, ns) or DM1 (DM1 Con: 54 ± 13% vs. DM1 RIPC: 53 ± 10%, ns).

### 2.3. Cardiac Function

Hemodynamic data from part 1 and part 2 are demonstrated in Table 2 and Table 3, respectively. There were no statistical differences measured between groups at baseline and during ischemia or reperfusion. LVDP and coronary flow significantly decreased during reperfusion compared to baseline within each group. For all in vivo experiments of the study, hemodynamic data demonstrated no significant differences between groups.

## 3. Discussion

The main findings of our current study demonstrate that (1) RIPC leads to the release of humoral factors under normoglycemia and DM1 that confer cardioprotection in healthy hearts, (2) hyperglycemic plasma possibly contains cardioprotective properties, and (3) infarct-size-reducing effects of RIPC are completely abolished in DM1 and HG myocardium.

Comorbidities, specifically diabetes mellitus alongside hyperglycemia, have been discussed extensively as a main factor interfering with beneficial cardioprotective effects by RIPC in clinical trials. Previous experimental studies have shown that diabetes—both type 1 and 2—and hyperglycemia lead to a loss of cardioprotection by several ischemic [18,19] and pharmacological [20] conditioning strategies. These findings were also supported in clinical studies, where beneficial effects of ischemic preconditioning were fully abolished in patients suffering from diabetes [17]. Referring to cardioprotection by ischemic conditioning strategies and comorbidities, experimental studies have detected a negative influence of diabetes and hyperglycemia on different myocardial signaling pathways [18]. Ischemic preconditioning (IPC) is blocked by diabetes mellitus through activation of glycogen synthase kinase 3-beta (GSK3β), which is also a critical mediator for several pharmacological agents conferring cardioprotection [21]. Extensive evidence implicates that inhibition of GSK3β by different protein kinases is needed for suppression of mitochondrial permeability transition pore (mPTP) opening and, thus, protection against I/R injury [22]. Acute hyperglycemia inhibits phosphorylation of protein kinase B (Akt) as part of the cardioprotective signaling pathway, and thus abolishes infarct size reduction by IPC [23]. Furthermore, dysfunction of mitochondrial adenosine-triphosphate-dependent potassium (mK_ATP_) channels due to increased ATP levels under hyperglycemic conditions has been implied to block protective effects of IPC under diabetes [24].

However, ischemic conditioning stimuli are applied directly at the heart, while RIPC depends on humoral factors released after a stimulus at a remote organ or tissue. Thus, when addressing potential influencing factors on cardioprotection by RIPC, a distinction must be made between an impact on signaling cascades in the myocardium itself and release or transfer of protective stimuli to the heart. To this day, detailed information on the potential impact of comorbidities on RIPC is lacking. Baranyai et al. [25] demonstrated that acute hyperglycemia, independent of a preceding diabetes, abolished infarct size reduction by remote ischemic per-conditioning (RIPerC). Increased nitrative stress, as well as activation of the mechanistic target of rapamycin (mTOR) pathway—a main regulator of cardiac autophagy—were involved in hyperglycemia-induced loss of RIPerC. To our knowledge, our study is the first to investigate the influence of diabetes and hyperglycemia on the release of humoral factors by RIPC, as well as demonstrate a clear distinction between diseased myocardium and protective factors. Next to comorbidities, the anesthetic regimen—specifically propofol—has been discussed to block cardioprotection in clinical trials. Bunte et al. [26] showed that propofol had no influence on cardiac signaling but inhibited the release or transfer of humoral factors after RIPC. These findings, which are contrary to results on comorbidities and RIPC in our current study, underline the differential impact of the various possible confounding factors on cardioprotection. Results from our study match those from an in vivo animal study on humoral factors after RIPC by Pickard et al. [27]. The authors demonstrated that an intact afferent nerve system is needed for the release of protective factors by RIPC. This is further underlined in a clinical trial on diabetic neuropathy and RIPC, demonstrating that cardioprotection by released humoral factors after RIPC is only achieved with preserved neuronal pathways in patients suffering from diabetes mellitus [28].

While the results from our study demonstrate that humoral factor release is independent of diabetes mellitus and hyperglycemia but cardioprotection by RIPC is blocked in diseased myocardium, no conclusive statement on underlying mechanisms of this effect can be made. As mentioned above, several studies have demonstrated altered mechanisms under diabetes and hyperglycemia, including an increase in reactive oxygen species, decreased nitric oxide availability [29,30], and impaired mitochondrial function [31]—especially referring to mPTP regulation. Further, various protein kinases critically involved in cardioprotective signaling (phosphatidylinositol-3-kinase, protein kinase C, mitogen-activated protein kinases and Akt) are influenced by hyperglycemia in the progression of diabetes mellitus [32]. All these elements are integral players in cardioprotection by RIPC in healthy hearts [33]. Thus, it seems obvious that infarct size reduction by RIPC is abolished in diseased myocardium due to alteration of these mechanisms under diabetes and hyperglycemia. However, further research is needed investigating this topic in more detail.

Interestingly, our findings show that plasma taken from hyperglycemic animals might contain protective factors conveying cardioprotective effects. Healthy hearts subjected to I/R injury after treatment with plasma from hyperglycemic animals showed infarct sizes comparable to known protective stimuli [34]. Notably, this effect could not be intensified by applying hyperglycemic plasma from animals treated with RIPC. This suggests that acute hyperglycemia itself possibly leads to the release of protective factors into the blood, completely independent of an additional conditioning strategy. Increased insulin plasma levels in hyperglycemic animals might be a possible explanation. Previous studies have shown that elevated glucose levels lead to an enhanced secretion of insulin in animals [35] and humans [36]. It could be assumed that induction of acute hyperglycemia with a glucose bolus, as in our experimental in vivo setup, resulted in an insulin response, with elevated levels at the time of plasma collection. Application of insulin as a conditioning strategy has been shown to induce cardioprotection in vitro and in vivo by triggering myocardial signaling cascades [37]. In particular, a glucose–insulin–potassium (GIK) infusion has been advocated and routinely used in cardiac patients to protect against myocardial I/R injury. In 2011, a meta-analysis including 2113 patients showed that patients undergoing cardiac surgery had a significantly lower incidence of perioperative myocardial infarction when receiving GIK compared to the control [38]. Further GIK improved postoperative cardiac index and reduced length of ICU stay in respective patients. Interestingly, in a subgroup analysis on diabetic patients, results demonstrated that GIK without glucose control had no beneficial effects on the above-mentioned endpoints. In contrast, in nondiabetic patients, cardioprotective effects of GIK were completely independent of glucose control [38]. In line with these findings, Marfella et al. [39] analyzed the effects of tight glycemic control in relation with GIK on regenerative potential in ischemic myocardium. Results demonstrated improved regenerative potential in patients with tight glycemic control by insulin treatment, while GIK alone had no beneficial effects. Authors concluded that the cardioprotective effects of insulin might by abrogated by hyperglycemia [39]. As patients included in the treatment groups had diagnosed diabetes and/or diabetic HbA1c levels (8% or higher) [39], findings from Marfella and colleagues further strengthen the hypothesis that optimal glucose management—possibly independent of treatment regimens—is crucial to achieve myocardial protection in diabetic patients [40]. Results from our study are in line with the above-mentioned divergent findings on nondiabetic and diabetic patients. We demonstrated that application of hyperglycemic plasma, possibly containing increased insulin levels, induced significant infarct size reduction when transferred onto healthy (nondiabetic and nonhyperglycemic) hearts. In part 2, no protective effects by transfer of RIPC plasma were detected in diseased (hyperglycemic and/or diabetic) animals, which supports the presumption that hyperglycemia abrogates cardioprotective effects. However, we did not examine whether plasma from hyperglycemic animals transferred onto diseased hearts is able to exert myocardial infarction. Further research is needed to evaluate the influence of hyperglycemic control and insulin levels in the context of plasma transfer and cardioprotection.

There are a few limitations of our study that need to be addressed. While acute hyperglycemia irrespective of a pre-existing diabetic disease—as investigated in our experimental setup—can be found in patients undergoing cardiac surgery, sudden induction of diabetes mellitus by streptozotocin does not fully represent the physiological scenario in humans. In the clinical setting, a prolonged onset and different stages are characteristic for diabetes mellitus [18]. We mainly focused on type 1 diabetes, meaning a total loss of insulin due to destruction of pancreatic β-cells by streptozotocin [41]. Patients in the focus of cardioprotective strategies more commonly suffer from type 2 diabetes mellitus, characterized by insulin resistance. However, with progression of the disease, these patients also often develop insulin deficiency. Interestingly, changes in metabolism caused by high glucose levels and, hence, altered signaling mediators are similar in both types of diabetes mellitus [32]. Previous studies have shown that variations in diet and types of fat can interfere with the diabetic pathology [42]. Employing our experimental setup, we ensured that focus is placed on the hyperglycemic or diabetic condition itself, independent of influencing factors caused by a prolonged disease progression, diet, or a panoply of additional cardiovascular risk factors in diabetic animals [18]. Another limitation is the lack of insulin measurements after plasma sampling. Thus, while elevated insulin levels could be a possible explanation for cardioprotective properties of plasma from hyperglycemic animals, at this point, we can only speculate on this aspect, and further research is needed. Finally, when addressing the translation of results from experimental studies on cardioprotection into clinical trials, one main difference with our setup and most animal models needs to be considered. Patients with diabetes mellitus often suffer from a multitude of comorbidities, such as hypertension or dyslipidemia, known to be cardiovascular risk factors [43]. Therefore, previous studies [44] and reviews [45,46,47] have discussed a multifactorial therapy approach to achieve cardioprotection and improve outcome in patients suffering from myocardial infarction. This current study was designed to focus on one of the main comorbidities as a first step in further investigating diabetes in the context of cardioprotection without the influence and interaction of other diseases. However, future experimental studies should place focus on cardioprotective strategies in animals suffering from different comorbidities for better translation into clinical practice.

## 4. Materials and Methods

The study was approved by the local animal care and use committees (in vivo: North Rhine-Westphalia Office of Nature, Environment and Consumer Protection (LANUV), Germany, reference number: 84-02.04. 2015. A514; in vitro: Heinrich Heine University Duesseldorf, Germany, reference number: O27/12). Investigations were conducted according to the ‘Guide for the Care and Use of Laboratory Animals‘ published by the U.S. National Institute of Health [48]. All experiments, in vivo plasma sampling and in vitro Langendorff model, were performed on 2–3-month-old male Wistar rats, obtained from the breeding facility at the Central Animal Research Facility of the Heinrich Heine University Duesseldorf. Animals were housed in the Central Animal Research Facility of the Heinrich Heine University Duesseldorf and kept with access to water and standard laboratory chow ad libitum at a 12 h light/dark cycle. In vivo and in vitro experiments were started after a 7-day acclimatization period for each animal.

The experimental study is comprised of two main parts. Both parts included in vivo experiments and plasma sampling prior to in vitro experiments in isolated hearts (Langendorff model). Part 1 was designed to unravel the influence of diseased conditions, such as diabetes mellitus (DM) and hyperglycemia (HG), on humoral factor release after RIPC. In part 2, we investigated the impact of diseased myocardium itself on RIPC treatment. Animals included in this study were randomly assigned to the respective study groups in part 1 and 2.

### 4.1. In Vivo Experiments and Plasma Sampling

#### 4.1.1. Surgical Preparation and RIPC Protocol

Surgical preparation was performed as previously described in detail [49]. Animals were not fasted before anesthesia to rule out a possible influence of intermittent fasting on cardioprotection [50,51]. General anesthesia was induced in all animals by intraperitoneal (i.p.) injection of 80 mg/kg bodyweight pentobarbital (Narcoren, Merial, Germany). Rats were placed in supine position onto a heating plate for the surgical procedure. The body temperature was monitored throughout the whole experiments using a rectal probe for rodents. Animals were intubated by ventral cervical incision, preparation of the trachea, and placement of a 16-gauge endotracheal catheter for ventilation. Mechanical ventilation (85 bpm, 2.5 mL TV, 30% oxygen/70% nitrogen) was maintained throughout the in vivo experiment and monitored by blood gas analysis (BGA). Continuous application of pentobarbital (40 mg/kg bodyweight/h) was used for general anesthesia. After successful intubation, both the right carotid artery and the left jugular vein were cannulated for hemodynamic measurements (arterial line) and application of glucose, saline and/or pentobarbital infusion (central venous line).

For implementation of RIPC maneuver, modified blood pressure cuffs were placed around both hind limbs. RIPC was induced by 4 cycles of 5 min bilateral hind-limb ischemia—via inflating blood pressure cuffs to 200 mmHg—alternating with 5 min of reperfusion. Control animals received the same surgical preparation and treatment, but without inflation of blood pressure cuffs. Sufficient induction of ischemia was verified by occurrence of visual limb cyanosis. In contrast, deflation of pressure cuffs immediately led to visual reperfusion as seen by returning of the limb to normal (preischemic) skin color. Five minutes after final reperfusion, a total of 10 mL arterial blood was collected, centrifugated for plasma separation, and stored at −80 °C for further use in vitro.

#### 4.1.2. Induction of DM1 and HG

Diabetes mellitus type 1 was induced in healthy male Wistar rats by a single intraperitoneal injection of streptozotocin (65 mg/kg) 21 days prior to in vivo experiments [52,53]. Streptozotocin was dissolved in 50 mM sodium citrate buffer, combined with citric acid to ensure pH levels of 4.5 [54]. Control animals were treated with intraperitoneal injection of sodium citrate buffer as vehicle. Weekly controls of blood sugar levels were performed in all animals using the Accu-Check Aviva (Roche). Streptozotocin-induced DM1 is a well-established pharmacological protocol in rats, which has been shown to selectively destroy pancreatic β-cells, leading to glucotoxicity and insulin deficiency [52,53]. Animals develop severe DM1, with blood glucose levels between 250 and 600 mg/dL as early as 24–72 h after injection [41]. In our study, successful induction of diabetes was confirmed by the presence of glucose values above 300 mg/dL.

Hyperglycemia was induced during in vivo experiments by intravenous administration of 40% glucose solution via cannulation of the jugular vein. Perfusion with glucose solution was started 5 min before the RIPC maneuver in healthy male Wistar rats under general anesthesia. After an initial bolus of 0.5 mL G40, a continuous perfusion of 1.5–3.5 mL/h G40 was applied during the experiment. Blood glucose levels were determined before RIPC, as well as throughout the whole in vivo experiments, and perfusion rate was adjusted accordingly to ensure hyperglycemia with glucose values above 300 mg/dL.

### 4.2. In Vitro Experiments and Plasma Transfer

#### 4.2.1. Surgical Preparation

Hearts from healthy or diseased male Wistar rats were randomly assigned to one of the experimental groups in part 1 or 2 of the study. The procedure was carried out as described previously [55]. Animals were anesthetized by i.p. injection of pentobarbital (Narcoren, Merial, Germany) (80 mg/kg body weight). Subsequent to decapitation, hearts were excised via thoracotomy and placed onto a Langendorff System under constant pressure (80 mmHg) and temperature (37 °C). Pressure-controlled perfusion was achieved with Krebs–Henseleit buffer (118 mM NaCl, 4.7 mM KCl, 1.2 mM MgSO_4_, 1.17 mM KH_2_PO_4_, 24.9 mM NaHCO_3_, 2.52 mM CaCl_2_, 11 mM glucose, and 1 mM lactate), enriched with a mix of 95% O_2_ and 5% CO_2_. For continuous hemodynamic measurements, a saline-filled balloon was inserted into the left ventricle with a set end-diastolic pressure of 4–6 mmHg. All measurements were digitized at a sampling rate of 500 Hz (PowerLab/8SP, ADInstruments Pty Ltd., Castle Hill, Australia) and recorded using Labchart 8.0 for Windows (ADInstruments Pty Ltd., Castle Hill, Australia). Hemodynamic data included heart rate, left ventricular end-systolic pressure (LVESP), left ventricular end-diastolic pressure (LVEDP), and left ventricular developed pressure (LVDP) (calculated as LVESP-LVEDP). For additional reference of myocardial damage, maximal contracture during ischemia, as well as the respective time point, was analyzed for each experiment. Next to hemodynamic data, coronary flow and glucose levels were measured throughout the experiments. Coronary flow was measured by collecting perfusate effluent for one minute each (expressed as milliliter per minute).

After 60 min of reperfusion, hearts were collected and cut into 8 transverse 2 mm slices per heart for subsequent staining with 0.75% triphenyltetrazoliumchloride (TTC) solution. A blinded, experienced investigator [56] determined the size of the infarcted area by planimetry using SigmaScan Pro5 software (Version 5.0.0). Infarct size is expressed as percentage of infarct area per total area of the left ventricle.

#### 4.2.2. Langendorff Protocol

All hearts underwent 15 min of adaption period, 33 min of global ischemia, followed by 60 min of reperfusion. Preconditioning (PC) was achieved by administration of undiluted plasma via a syringe pump (Perfusor Space, B. Braun, Melsungen, Germany) at an infusion rate of 1% of the coronary flow. Plasma was not dissolved but applied in pure form over 10 min, before induction of global ischemia.

#### 4.2.3. Induction of HG

Acute hyperglycemia in hearts included in part 2 was induced by administration of an additional 11 mM glucose solution during the in vitro protocol. Perfusion with glucose was started five minutes prior to preconditioning and stopped with the onset of global ischemia. By combining this solution and perfusion with Krebs–Henseleit buffer (already consisting of 11 mM glucose), a total of 22 mM glucose concentration was achieved at the heart. Ensuring hyperglycemic conditions, glucose levels (mg/dL) were determined in collected effluent continuously throughout each experiment using a blood gas analyzer (ABL800Flex Plus, Radiometer, Krefeld Germany). The employed protocol was taken from a previous own study, ensuring hyperglycemia with glucose values above 300 mg/dL [34].

#### 4.2.4. Part 1: “Release of Humoral Factors”—Plasma Transfer: Diseased → Healthy

**Figure 3:**
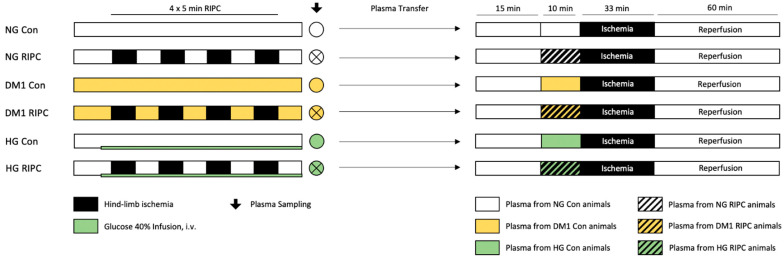
Experimental protocol part 1. Con = control; RIPC = remote ischemic preconditioning; NG = normoglycemia; DM1 = diabetes mellitus type 1; HG = hyperglycemia.

Plasma sampled from normoglycemic (NG) or diseased (DM1 or HG) animals—with or without prior RIPC treatment in vivo—was transferred onto naïve hearts from healthy, male Wistar rats, as shown in Figure 3.

**Control (Con):** Hearts were perfused with plasma collected in vivo from NG, DM1, or HG animals without RIPC treatment.

**Remote ischemic preconditioning (RIPC):** Hearts received plasma collected in vivo from normoglycemic (NG), DM1, or HG animals that underwent RIPC treatment.

#### 4.2.5. Part 2: “Influence of Diseased Myocardium”—Plasma Transfer: Healthy → Diseased

**Figure 4:**
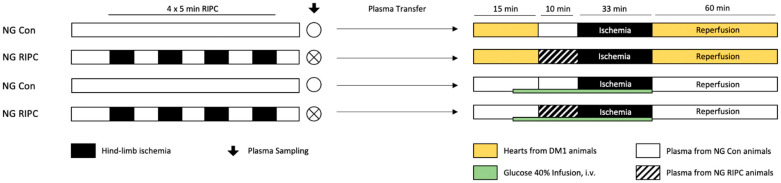
Experimental protocol part 2. Con = control; RIPC = remote ischemic preconditioning; NG = normoglycemia; DM1 = diabetes mellitus type 1; HG = hyperglycemia.

Plasma from healthy normoglycemic animals was collected in vivo with (RIPC) or without (Con) RIPC treatment and transferred onto diseased hearts (DM1 or HG) from male Wistar rats (Figure 4).

**Control (Con):** Plasma collected from normoglycemic in vivo Con animals was administered onto hearts from DM1 or HG rats.

**Remote ischemic preconditioning (RIPC):** Plasma collected from normoglycemic in vivo RIPC animals was administered onto hearts from DM1 or HG rats.

### 4.3. Statistical Analysis

The primary endpoint of our study was infarct size determination. Plasma taken from one animal in vivo was employed for one in vitro experiment, respectively. Therefore, the group size in the in vivo part generates from sample size calculation of infarct size determination in vitro. Detecting a 25% mean difference and a standard deviation of 16% in infarct size (power 80%, α < 0.05 (two-tailed)), a group size of n = 8 was revealed by sample size calculation (GraphPad StatMate™, GraphPad Software, San Diego, CA, USA) for each part of the study. Infarct size was analyzed by Student’s *t*-test. A two-way analysis of variance (ANOVA) and a Tukey post hoc test (GraphPad Software V7.01, San Diego, CA, USA) were performed for comparison of hemodynamic data between groups, as well as between different time points within groups. Baseline values were taken as a reference time point. Data are presented as mean ± standard deviation (SD) and changes are considered statistically significant if *p* < 0.05.

## 5. Conclusions

Taken together, our results suggest that the blockade of cardioprotection by comorbidities, such as DM1 and hyperglycemia, is caused by elements in the diseased myocardium itself, while these comorbidities seem to have no effect on the release of humoral factors after RIPC. These findings are of high importance in overcoming critical translation of conditioning strategies into the clinical setting in light of comorbidities. Further studies need to place a focus on clarifying blocked signaling cascades in diseased myocardium under diabetic and hyperglycemic conditions, as humoral factor release after RIPC is still effective under these comorbidities.

## Figures and Tables

**Table 1:** Weights and ischemic contracture.

		n	Body Weight (g)	Heart Weight Wet (g)	Heart Weight Dry (g)	Time of Max. Ischemic Contracture (min)	Level of Max. Ischemic Contracture (mmHg)
Part 1—plasma transfer diseased → healthy							
NG	Con	9	288 ± 16	1.25 ± 0.11	0.10 ± 0.02	15 ± 2	77 ± 11
	RIPC	7	285 ± 21	1.22 ± 0.09	0.09 ± 0.02	15 ± 1	73 ± 13
HG	Con	7	300 ± 20	1.26 ± 0.07	0.10 ± 0.01	16 ± 2	62 ± 13
	RIPC	8	299 ± 21	1.26 ± 0.10	0.09 ± 0.01	14 ± 1	74 ± 11
DM1	Con	7	288 ± 20	1.22 ± 0.11	0.10 ± 0.01	15 ± 2	73 ± 11
	RIPC	8	281 ± 13	1.21 ± 0.04	0.10 ± 0.01	16 ± 3	66 ± 14
Part 2—plasma transfer healthy → diseased							
HG	Con	8	283 ± 15	1.25 ± 0.07	0.11 ± 0.01	17 ± 1	99 ± 12
	RIPC	9	290 ± 15	1.25 ± 0.08	0.11 ± 0.01	19 ± 2	96 ± 15
DM1	Con	8	257 ± 39	1.15 ± 0.13	0.10 ± 0.01	20 ± 3	77 ± 9
	RIPC	8	246 ± 28	1.14 ± 0.14	0.10 ± 0.01	20 ± 2	74 ± 11

Data are mean ± SD, Con = control; RIPC = remote ischemic preconditioning; NG = normoglycemia; HG = hyperglycemia; DM1 = diabetes mellitus type 1.

**Table 2:** Hemodynamic variables from part 1 (plasma transfer diseased → healthy).

		Baseline	PC	Reperfusion	
				33 min	60 min
Heart Rate (bpm)					
NG	Con	312 ± 37	286 ± 46	205 ± 69	227 ± 75
	RIPC	301 ± 56	269 ± 52	265 ± 55	244 ± 51
HG	Con	288 ± 25	257 ± 36	224 ± 51	272 ± 34
	RIPC	303 ± 30	278 ± 39	229 ± 74	220 ± 72
DM1	Con	315 ± 28	311 ± 36	302 ± 55	236 ± 64
	RIPC	310 ± 23	274 ± 53	256 ± 52	229 ± 59
Left Ventricular Developed Pressure (mmHg)					
NG	Con	143 ± 15	132 ± 24	22 ± 12 *	28 ± 7 *
	RIPC	152 ± 20	132 ± 27 *	45 ± 9 *	46 ± 20 *
HG	Con	141 ± 22	123 ± 35	27 ± 13 *	34 ± 13 *
	RIPC	142 ± 18	135 ± 19	25 ± 11 *	34 ± 9 *
DM1	Con	146 ± 29	131 ± 25	17 ± 5 *	27 ± 11 *
	RIPC	132 ± 32	119 ± 32	31 ± 12 *	40 ± 12 *
Coronary Flow (ml/min)					
NG	Con	16 ± 3	14 ± 5	7 ± 3 *	6 ± 2 *
	RIPC	14 ± 3	11 ± 3 *	9 ± 3 *	7 ± 3 *
HG	Con	18 ± 3	13 ± 4 *	9 ± 1 *	7 ± 1 *
	RIPC	17 ± 5	16 ± 5	8 ± 3 *	7 ± 2 *
DM1	Con	15 ± 4	13 ± 3	7 ± 2 *	6 ± 2 *
	RIPC	13 ± 3	10 ± 2 *	7 ± 1 *	7 ± 2 *

Data are mean ± SD. Con = control; RIPC = remote ischemic preconditioning; NG = normoglycemia; HG = hyperglycemia; DM1 = diabetes mellitus type 1; PC = preconditioning. * *p* < 0.05 versus baseline.

**Table 3:** Hemodynamic variables from part 2 (plasma transfer healthy → diseased).

		Baseline	PC	Reperfusion	
				33 min	60 min
Heart Rate (bpm)					
HG	Con	324 ± 24	283 ± 34	267 ± 96	255 ± 92
	RIPC	280 ± 16	267 ± 22	249 ± 71	223 ± 47
DM1	Con	258 ± 40	230 ± 28	238 ± 62	222 ± 39
	RIPC	253 ± 30	236 ± 22	224 ± 44	221 ± 52
Left Ventricular Developed Pressure (mmHg)					
HG	Con	122 ± 34	94 ± 20	31 ± 12 *	35 ± 12 *
	RIPC	127 ± 30	110 ± 21	40 ± 11 *	48 ± 11 *
DM1	Con	131 ± 20	114 ± 18	34 ± 7 *	36 ± 20 *
	RIPC	116 ± 28	106 ± 29	39 ± 9 *	40 ± 8 *
Coronary Flow (ml/min)					
HG	Con	13 ± 3	10 ± 2	5 ± 1 *	5 ± 1 *
	RIPC	11 ± 3	10 ± 1	6 ± 2 *	6 ± 2 *
DM1	Con	11 ± 2	8 ± 2	7 ± 2 *	7 ± 2 *
	RIPC	10 ± 2	8 ± 2	6 ± 1 *	6 ± 1 *

Data are mean ± SD. Con = control; RIPC = remote ischemic preconditioning; HG = hyperglycemia; DM1 = diabetes mellitus type 1; PC = preconditioning. * *p* < 0.05 versus baseline.

## Data Availability

Not applicable.

## Supplementary Materials

The following are available online at https://www.mdpi.com/article/10.3390/ijms22168880/s1.
