# Supplementary material for: Influence of Hyperglycemia and Diabetes on Cardioprotection by Humoral Factors Released after Remote Ischemic Preconditioning (RIPC)
Source: Int J Mol Sci. 2021 Aug 18;22(16):8880. doi: 10.3390/ijms22168880 (PMC8396298; doi:10.3390/ijms22168880)
Supplement: Supplementary file 1 [file ijms-22-08880-s001.zip › ijms-1326931-supplementary.pdf]

Table S1. Body weight (*in vivo* plasma sampling).

		n	body weight (g)
Part 1 - plasma sampling diseased			
NG	Con	9	316 ± 46
	RIPC	7	325 ± 51
HG	Con	7	304 ± 19
	RIPC	8	322 ± 32
DM1	Con	7	248 ± 37
	RIPC	8	273 ± 53
Part 2 - plasma sampling healthy			
NG	Con	16	304 ± 24
	RIPC	17	307 ± 23

Data are mean±SD, Con = Control; RIPC = Remote Ischemic Preconditioning; NG = Normoglycemia; HG = Hyperglycemia; DM1 = Diabetes Mellitus Type 1.

Table S2. Body weight progression DM1.

		n	body weight (g) injection	body weight (g) week 1	body weight (g) week 2	body weight (g) week 3
Part 1 - plasma sampling in vivo						
DM1	Con	7	203 ± 32	214 ± 17	244 ± 26	248 ± 37
	RIPC	8	213 ± 13	239 ± 31	264 ± 46	273 ± 53
Part 2 - plasma transfer in vitro						
DM1	Con	8	198 ± 10	222 ± 19	247 ± 33	257 ± 39
	RIPC	8	203 ± 19	221 ± 19	245 ± 27	246 ± 28

Data are mean±SD, Con = Control; RIPC = Remote Ischemic Preconditioning; DM1 = Diabetes Mellitus Type 1.
